# Implications of Rho GTPase signaling in cancer immunotherapy

**DOI:** 10.1042/BST20260485

**Published:** 2026-06-24

**Authors:** Mingjun Cai, Fukun Guo, Yi Zheng

**Affiliations:** 1 Graduate Program of Development, Stem Cells, and Regenerative Medicine, University of Cincinnati College of Medicine, 3333 Burnet Avenue, Cincinnati, OH 45229, U.S.A.; 2 Division of Experimental Hematology and Cancer Biology, Cancer and Blood Diseases Institute, Children’s Hospital Medical Center, and Department of Pediatrics, University of Cincinnati College of Medicine, 3333 Burnet Avenue, Cincinnati, OH 45229, U.S.A.

**Keywords:** CAR-T therapy, immune checkpoint inhibitor, immunotherapy, Rho GTPase signaling, tumor microenvironment

## Abstract

Cancer immunotherapy, including immune checkpoint inhibitors (ICIs) and chimera-antigen receptor (CAR)-T cell therapy, has achieved substantial clinical success. However, response rates remain limited in many patients due to tumor-intrinsic immune evasion and immune cell dysfunction within the tumor microenvironment (TME). Rho family small GTPases are key signaling regulators of cytoskeletal dynamics, intracellular trafficking, transcription, and metabolism in cancers. Emerging evidence implicates Rho GTPase signaling in mediating immunotherapy efficiency through its context-dependent functions. Individual Rho GTPases modulate immunotherapy responses in tumor cells and various immune cells through actomyosin-mediated chemotaxis, cell junctions, cell polarity, and gene/epigenetic networks, among other pathways. The present review summarizes both the direct evidence linking Rho GTPases in tumor cells to immunotherapy responses and the indirect role of the selective Rho GTPase signaling network in various immune cells, with a focus on the recent progress in understanding the molecular mechanisms and associated outcomes of the ICIs and CAR-T cell therapies. We highlight current knowledge gaps at the intersection of Rho GTPase biology and cancer immunology and discuss therapeutic implications, proposing that selective modulation of specific Rho GTPase signaling pathways in tumor or TME immune cells represents a promising strategy to improve immunotherapy efficiency.

## Introduction

Cancer immunotherapy is a therapeutic strategy that boosts patients’ immune systems to recognize and eliminate cancer cells [1]. It comprises a broad spectrum of approaches, including immune checkpoint inhibitors (ICIs), adoptive cell therapies (such as CAR-T, CAR-NK, and TCR-T cells), targeted antibodies (such as monoclonal antibodies, bispecific antibodies, and antibody–drug conjugates), cancer therapeutic vaccines, and oncolytic viruses [2,3]. Among these, ICIs and CAR-T therapies have emerged in recent years as the most impactful FDA-approved immunotherapeutic strategies, with their high efficiency as evidenced by durable response, long-term survival benefit, and high objective response rate [4–8]. Although these therapies have seen remarkable clinical success, a substantial proportion of patients fail to respond [3], highlighting the need to identify mechanisms of tumor-immune interactions for improvement.

Rho family GTPases are central regulators of cell cytoskeletal dynamics and polarity. Beyond these classical functions, Rho GTPases have emerged as critical signaling hubs that control vesicle trafficking, transcriptional regulation, and cell metabolism [9,10]. Dysregulation of Rho GTPase signaling is closely related to cancer, contributing to various aspects of cancer initiation, progression, metastasis, and therapy resistance [9]. Importantly, like the cases of oncogenic RAS, Rho GTPases and their downstream effectors are critical for immune cell development and TME modulation [11–13]. However, while there has been a solid understanding of RAS-regulated tumor microenvironment contributing to tumorigenesis and therapy response [14], the role of Rho GTPases in regulating tumor immunity and immunotherapy response has only begun to emerge.

The Rho GTPase family consists of 20 members, including classic members such as RHOA, RAC1, and CDC42, as well as non-classical members [15]. Besides the extensively studied roles of RHOA, RAC1, and CDC42, existing evidence suggests that some non-classical Rho GTPases also contribute to cancer progression and immune cell function. For example, RhoC is involved in tumor invasion and metastasis [16], and a RhoC-targeting cancer vaccine has been shown to induce potent and durable T cell immunity in patients with prostate cancer [17]. In T cells, the RhoH facilitates the recruitment of Lck and ZAP70 to phosphorylated CD3ζ, thereby enhancing T cell receptor (TCR) signaling, a pathway closely associated with T cell activation and exhaustion [18]. Given the limited studies of non-classical Rho GTPases in cancer immunotherapy to date, the present review primarily focuses on the role of classical Rho GTPases, i.e., RHOA, RAC1, and CDC42. We summarize the fundamental signaling functions of these Rho GTPases in the context of recent progress and limitations of major cancer immunotherapeutic strategies and present a forward-looking discussion on how our understanding of Rho GTPase signaling may evolve in cancer immunotherapy.

## Rho GTPase signaling mechanism and function

**Figure 1:**
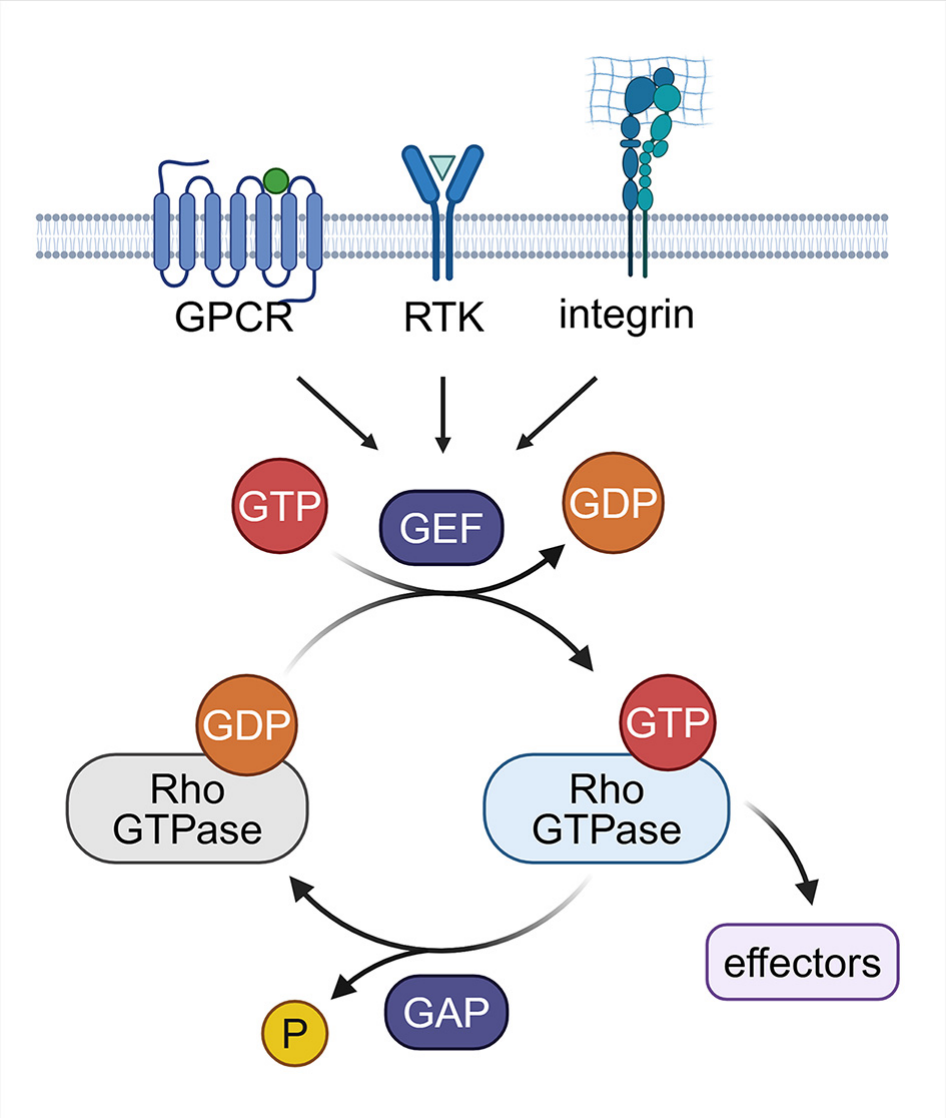
Rho GTPase signaling nodules Rho GTPases function as molecular switches that integrate upstream signals from surface receptors, cycling between inactive GDP- and active GTP-bound states under the control of guanine nucleotide exchange factor (GEF) and GTPase-activating protein (GAP). In their GTP-bound state, Rho GTPases activate effector proteins to regulate intracellular signaling.

The classic Rho family GTPases including RHOA, RAC1, and CDC42 are molecular switches cycling between an inactive GDP-bound state and an active GTP-bound state, a process tightly regulated by guanine nucleotide exchange factors, which promote activation; GTPase-activating proteins, which accelerate GTP hydrolysis; and guanine nucleotide dissociation inhibitors, which sequester inactive GTPases in the cytosol [10,19–21] (Figure 1). Through this regulatory network, Rho GTPases ensure spatially and temporally precise signaling, allowing cells to dynamically respond to biochemical and mechanical cues during development, homeostasis, and disease [22–25].

Active Rho GTPases interact with a number of downstream effectors to transduce signals leading to diverse biological responses [19]. For example, RHOA activates Rho-associated coiled-coil-containing protein kinase (ROCK) 1/2 and formin family members such as mDia, driving actomyosin contractility, stress fiber assembly, and focal adhesion maturation, thereby regulating cellular tension and mechanical force generation [20]. RAC1 activates p21-activated kinases (PAKs) and the WAVE regulatory complex, which in turn stimulates Arp2/3-mediated actin nucleation to promote lamellipodia formation, membrane protrusion, and adhesion turnover [20,26,27]. CDC42 engages effectors including WASP, PAK, and the Par6–aPKC polarity complex to coordinate filopodia formation, cell polarity establishment, and asymmetric cytoskeletal organization. Detailed cell signaling mechanisms of individual Rho GTPases have been extensively discussed elsewhere in several excellent reviews [9,28–30].

## Rho GTPase in immune checkpoint inhibitor therapy

### Immune checkpoint inhibitor therapy and its challenges

Immune checkpoints are regulatory molecules of immune cells that prevent inappropriate immune-mediated damage to healthy cells [1]. Malignant cells frequently hijack them to evade T cell-mediated surveillance and destruction [1]. ICIs are therapeutic agents that block these inhibitory signals to restore T cell activity and enhance antitumor responses [1,31,32]. However, the efficiency of ICI monotherapy is often constrained by primary or acquired resistance in substantial patient populations [33].

Factors limiting efficiency and clinical response to ICIs include both tumor-intrinsic mechanisms and those of the immunosuppressive tumor microenvironment (TME), which act cooperatively to restrain antitumor immunity. Tumor-intrinsic mechanisms primarily involve defects in tumor immunogenicity and immune recognition. Tumors with a low tumor mutation burden or low expression level of neoantigen often fail to elicit robust T cell responses [34]. In addition, tumor cells can evade immune surveillance through diverse genetic and epigenetic alterations [35], and the altered immune checkpoint expression is also a determinant of ICI efficiency. An immunosuppressive TME facilitates ICI resistance by creating barriers that impair T cell priming, infiltration, and function [36]. Accumulation of immunosuppressive Tregs, myeloid-derived suppressor cells, and M2-like tumor-associated macrophages suppresses effector T cell activity through inhibitory cytokines such as TGF-β and IL-10 [36]. Abnormal tumor vasculature, dense stroma, and altered chemokine signaling restrict T cell trafficking, leading to immune ‘cold’ tumors [36–38]. Metabolic constraints, including hypoxia, nutrient depletion, and suppressive metabolite (such as adenosine, lactate, etc.) accumulation, can further promote effector T cell dysfunction and exhaustion [39–42].

### Tumor intrinsic regulatory role of Rho GTPases

**Figure 2:**
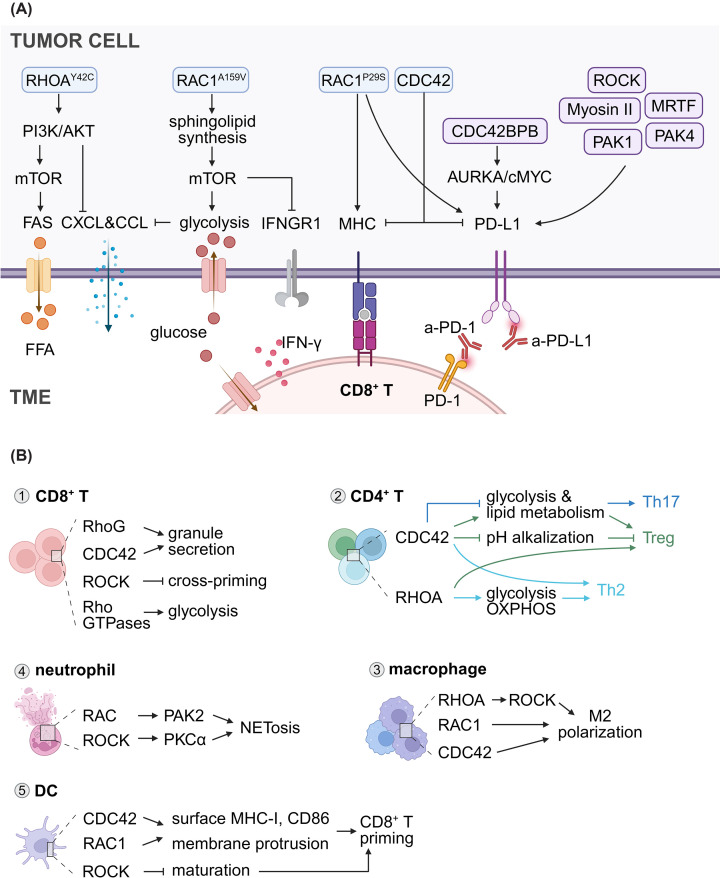
Rho GTPase signaling is involved in ICI efficiency (**A**) Individual tumor-intrinsic Rho GTPases and downstream effectors shape the immune microenvironment and tumor-CD8^+^ T cell interactions to affect ICI efficiency via distinct mechanisms. (**B**) Rho GTPases and downstream effectors regulate CD8^+^ T cell cytotoxic function, CD4^+^ T cell differentiation, macrophage polarization, NETosis, and dendritic cell (DC) maturation, thereby modulating anti-tumor immune responses and ICI efficiency.

Recent evidence shows that aberrant Rho GTPase signaling in cancer cells can affect tumor responsiveness to immunotherapies in a tumor type-dependent manner (Figure 2A). The RHOA^Y42C^ mutation represents a notable example of the complexity of Rho GTPase signaling in cancer. In large B-cell lymphomas, RHOA appears to function as a tumor suppressor. The biochemical analysis indicated that the RHOA^Y42C^ mutant exhibits a reduced active form, resulting in loss of PTEN-mediated repression of AKT, a tumor-suppressive pathway in large B-cell lymphoma [43–46]. In this context, Y42C, as a loss-of-function mutation, promotes tumorigenesis through AKT activation. However, further experimental data suggest that Y42C exhibits gain-of-function phenotypes in gastric cancer cells, including enhanced proliferation and stress fiber formation [47–49]. And gain-of-function RHOA^Y42C^ in gastric cancer cells confers resistance to anti-PD-1 monotherapy by activating the PI3K-AKT-mTOR signaling axis in cancer cells, which up-regulates fatty acid synthesis, leading to the accumulation of free fatty acids in the TME to promote Treg expansion and suppress anti-tumor immunity. In parallel, the up-regulated PI3K-AKT signaling also inhibits IRF1-regulated CXCL10 and CXCL11 expression, resulting in reduced recruitment of CD8^+^ effector T cells [13]. These findings highlight that tumor cell-intrinsic Rho GTPase signaling effects need to be interpreted in the tumor type-specific context.

Tumor-derived RAC1^A159V^ gain-of-function mutation also renders colon cancers and melanoma resistant to anti-PD-1 therapy via creating an immunosuppressive ‘cold’ TME. Mechanistically, RAC1^A159V^ up-regulates glycosphingolipid biosynthesis to activate mTORC1 signaling in tumor cells, which in turn increases glycolysis to deprive glucose in the TME critical for CD8^+^ effector T cell function; decreases CCL and CXCL chemokine production to impair immune cell infiltration; and decreases IFNGR1 expression to shield RAC1^A159V^ tumor from IFN-γ-mediated immune attack [12]. Different from RAC1^A159V^ mutation, RAC1^P29S^ gain-of-function mutation sensitizes YUMM1.7 melanoma to anti-PD-1 treatment [50]. This is possibly because RAC1^P29S^ up-regulates PD-L1 (programmed death-ligand 1) and MHC-I expression on tumor cells, and overexpressed RAC1^P29S^ increases infiltration of CD8^+^ T cell, and CD11c^+^ myeloid cell infiltration in TME [50,51].

CDC42 has been identified as a negative regulator of ICI efficiency. Clinical data demonstrate that tumors harboring CDC42 gene set mutations, which impair CDC42 signaling, exhibit significantly higher tumor mutational burden, neoantigen loads, and MHC-I/II expression compared with CDC42 wild-type tumors, which is indicative of better ICI outcomes. These mutant tumors also showed up-regulated immune checkpoints (including PD-1, PD-L1, and CTLA-4) and increased immune cell infiltration potentially driven by increased chemokine expression, thereby making tumors more sensitive to ICIs. Supporting these clinical observations, inhibition of CDC42 with ML141 in mouse model markedly reduced tumor growth compared with anti-PD-1 monotherapy (tumor growth inhibition rate 86% versus 38%) and increased immune cell infiltration in the TME [52]. CDC42 in tumors has also been shown to prevent oncogene-transformed murine embryonic fibroblasts from CD8^+^ T cell-induced cell apoptosis by activating MAPK-ERK signaling and post-transcriptionally stabilizing the antiapoptotic protein Bcl-2 [53]. Collectively, these findings provide strong evidence that CDC42 signaling mediates immune evasion and represents a targetable biomarker of ICI resistance. In addition, serum CDC42 level also becomes a biomarker in predicting ICI response in hepatocellular carcinoma and cervical cancer patients [54,55].

The role of tumor-intrinsic Rho GTPases in ICI response is further supported by multiple investigations focusing on Rho GTPase downstream effectors. In breast cancer cells, RHOA downstream effector ROCK phosphorylates moesin, which in turn competitively binds to the E3 ubiquitin ligase SPOP to prevent PD-L1 degradation [56]. ROCK also helps mobilize the internally stabilized PD-L1 on the tumor cell surface, although the underlying mechanism remains incompletely defined [57]. Consistent with these findings, ROCK downstream MRTF and myosin II pathways in melanoma correlate with elevated tumor cell surface PD-L1 expression, reduced infiltration of CD8^+^ T cells and B cells, and increased accumulation of immunosuppressive M2 macrophages as well as decreased ICI efficiency via regulating chemokine and cytokine secretion, collectively supporting an immune-resistant role of Rho signaling within tumor cells [58–60]. Notably, this paradigm is context-dependent, as the RHOA effector PKN2 in colon cancer cells suppresses M2 macrophage polarization by inhibiting ERK1/2-dependent induction of IL-4 and IL-10, highlighting functional divergence among Rho downstream pathways [61]. CDC42- and RAC1-associated effector kinases have also emerged as important modulators of tumor immunity. Tumor-intrinsic CDC42 effector CDC42BPB was identified as a resistance determinant for anti-PD-1 therapy in triple-negative breast cancer, where it promotes PD-L1 expression via AURKA-cMYC signaling axis [62]. Furthermore, PAK family kinases, downstream effectors of both RAC1 and CDC42, serve as critical TME modulators. Specifically, PAK1 in pancreatic cancer correlates with robust PD-L1 expression [63]. PAK4 in melanoma cells enforces resistance to anti-PD-1 by engaging WNT/β-catenin signaling, which suppresses dendritic cell recruitment, limits T cell infiltration, and sustains high PD-L1 abundance on tumor cells [64–66]. Furthermore, ERK1/2 signaling that functions downstream of PAK drives recruitment of M2 macrophages via enhanced secretion of the CCL2 and reinforces an immunosuppressive tumor microenvironment in glioblastoma [67].

### Rho GTPases in tumor immune microenvironment

Apart from tumor-intrinsic effects, Rho GTPases are broadly expressed in immune cells and critically shape the TME, a central determinant of immunotherapy responsiveness (Figure 2B). Multiple immune populations, including CD8^+^ effector/cytotoxic T cells, CD4^+^ helper T (Th) cell subsets, regulatory T cells (Tregs), macrophages, neutrophils, and DCs, collectively regulate anti-tumor immunity and ICI therapeutic outcomes [68,69]. Consistent with this concept, CDC42 expression in peripheral blood mononuclear cells has been identified as a predictive biomarker for anti-PD-1 response in colorectal cancer patients [70].

Rho GTPases in T cells regulate cytoskeletal remodeling, membrane domain organization, and T cell polarity, which are necessary for T cell chemotaxis and immune synapse formation [71,72]. In CD8^+^ effector T cells/cytotoxic T cell lymphocytes (CTLs), the major effector immune cell type in ICI therapy, deficiency of RhoG and CDC42 impairs cytotoxicity via inhibiting cytotoxic granule secretion [73,74]. Rho GTPase-mediated metabolic reprogramming, particularly enhanced glycolysis, can support CD8^+^ T cell proliferation and effector function, although direct evidence in the context of ICIs remains limited [75,76]. Collectively, these findings highlight an essential role for Rho GTPase signaling in sustaining effective tumor cell killing. However, a recent study reported that pharmacological inhibition of ROCK increases cross-priming of CD8^+^ T cells, promotes durable systemic immune memory, and suppresses tumor growth in a CD8^+^ T cell-dependent manner, underscoring the context- and pathway-specific effects of Rho GTPase signaling in immunotherapy [77]. Rho GTPases also govern CD4^+^ T cell plasticity and differentiation to affect ICI efficiency. CDC42 serves as a central regulator of Th17/Treg balance by suppressing Th17 differentiation and promoting Treg differentiation and stability via regulating glycolysis and lipid metabolism [78]. Treg cell-specific heterozygous deletion or pharmacological inhibition of CDC42 leads to Treg instability and increases anti-PD-1 efficiency via WASP-GATA3-CAI-mediated pH changes [79]. Similarly, heterozygous RHOA deletion in Treg cells induces Treg cell plasticity, thereby enhancing the accumulation of anti-tumor effector T cells within the TME [80]. Both CDC42 and RHOA have also been reported to promote immunosuppressive Th2 differentiation [81–83].

In myeloid cells, Rho GTPases predominantly support immunosuppressive phenotypes. In macrophages, RHOA-ROCK signaling promotes M2 polarization while suppressing pro-inflammatory M1 differentiation [84,85]. Up-regulation of RHOA, RAC1, and CDC42 facilitates filopodia formation and activates M2 polarization-related signaling pathways, skewing macrophages toward tumor-supportive states [86]. In neutrophils, RAC-PAK2 promotes histone H3 citrullination, and ROCK signaling induces PKCα nuclear translocation to drive neutrophil extracellular trap (NET) formation, which protects tumor cells from CD8^+^ T cell cytotoxicity, and it has been confirmed that inhibition of NETs significantly improves anti-PD-1 responses [87–90]. Rho GTPases are important for DC adherence, antigen presentation, migration, chemotaxis, and endocytosis [91]. CDC42 and RAC1 in DCs control the actin-dependent transport of immunostimulatory molecules to the cell surface as well as polarized membrane extensions toward T cells for efficient T cell priming [92,93]. However, pharmacologic ROCK inhibition was shown to enhance tumor phagocytosis, DC maturation, and T cell priming, thereby shifting tumors toward an immune-permissive state that could promote immunotherapy responsiveness [77].

Together, these findings indicate that Rho GTPase signaling exerts tumor type-dependent and sometimes opposing roles in regulating ICI response across tumor and immune cell compartments. Consequently, consideration of therapeutic targeting in ICI therapy will likely depend on tumor type, mutation status, and TME immune cell context, underscoring the need for devising context-specific approaches, including tumor and immune cell type-selective modulation.

## Rho GTPases in chimera-antigen receptor-mediated cell therapies

### CAR-T cell therapy and its limitations

CAR-T cell therapy is a FDA-approved adoptive cell therapy in which T cells are genetically engineered to express a chimera-antigen receptor (CAR) structure that enables targeted killing of cancer cells [94]. Despite the remarkable clinical success of CAR-T cell therapy in hematologic malignancies, its efficiency in solid tumors has remained limited due to additional biological and physical barriers that impede CAR-T cell trafficking, infiltration, and persistence in the TME [95,96].

In both hematologic malignancies and solid tumors, a major limitation of CAR-T therapy is T cell exhaustion, a dysfunctional T cell state characterized by impaired cytokine production, reduced proliferative capacity, and sustained expression of inhibitory receptors [97,98]. Current clinical CAR-T therapy depends on *ex vivo* manufacturing that often results in exhaustion and reduced stemness, with compromised CAR-T cell persistence [97,99,100]. T cell aging/senescence is another key limitation on CAR-T cell efficiency [101,102]. Recent groundbreaking studies have found that cancer itself accelerates host T cell ‘aging’ by transcriptional and epigenetic reprogramming that mirrors physiological aging, and subsequent *ex vivo* expansion of CAR-T cells may further drive cell culture-induced ‘aging’ by inducing DNA hypermethylation at specific sites [103,104].

### Potential role of Rho GTPases in CAR-T cell therapy

Loss of RHOA in large B-cell lymphomas is associated with poor response to CD19 CAR-T cell therapy [105], and constitutively active RAC1^V12^ mutation in CD33 CAR-T cells promotes CAR-T cell migration, residence, and cytotoxicity in bone marrow to suppress leukemia better [106]. Although this emerging evidence links Rho GTPase causally to CAR-T therapy response, studies of T cell intrinsic roles of Rho GTPases in determining CAR-based therapeutic outcomes remain limited. Current insights are mostly derived from studies of naïve T cell signaling, which implicate Rho GTPases in key regulatory processes but provide limited definitive roles in engineered CAR-T or CAR-NK cells in tumor-killing settings. As such, discussion of this area is more forward-looking, aimed at providing a conceptual framework to generate testable hypotheses.

**Figure 3:**
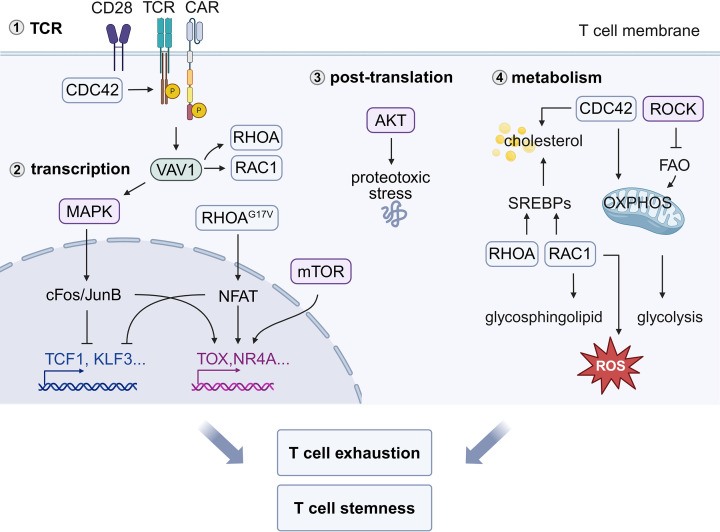
Schematic models of Rho GTPase signaling in T cell exhaustion and stemness In the context of CAR expression, individual Rho GTPases and their downstream effectors mediate TCR/CD28 signaling, gene transcription networks, post-translational protein homeostasis, and cellular metabolism, thereby contributing to T cell exhaustion or stemness in the long term.

The severity of T cell exhaustion is dependent on the strength and persistence of TCR signaling [107]. The Rho GTPases can regulate actin cytoskeletal remodeling, immune synapse formation, and TCR microcluster dynamics, thereby shaping TCR signal amplification, termination, and duration [108–111]. For example, CDC42 optimizes TCR signal acquisition through the organization of actin-dependent microvilli for TCR engagement and clustering and also modulates signal duration via clathrin-independent TCR endocytosis [109,110] (Figure 3). Beyond TCR regulation, RAC1 and RHOA could be activated downstream of TCR engagement via VAV1-dependent guanine nucleotide exchange activity [112,113].

Transcriptionally, Rho GTPase signaling axes converge on transcription factors that govern exhaustion-associated CAR-T cell fate decisions (Figure 3). RHOA^G17V^ has been shown to enhance NFAT-dependent transcription by promoting p300 histone acetyltransferase activity [114]. Under conditions of chronic TCR stimulation lacking appropriate costimulatory signals, NFAT becomes uncoupled from AP-1, inducing exhaustion-associated factors (e.g., TOX and NR4A) while suppressing memory and stemness regulators such as TCF1 and KLF3, leading to exhaustion [115,116]. In addition, more studies have linked Rho GTPase downstream effectors to the T cell or CAR-T cell fate and function. MAPK signaling, activated downstream of TCR or CAR signaling via VAV1-RAS, induces CAR-T cell exhaustion via c-Fos-JunB axis, restrains the transcriptional bias toward memory/naïve-like state, and impairs anti-tumor function [117–119]. Similarly, T cell-intrinsic mTOR signals are required for the differentiation of stem-like or progenitor exhausted T cells into terminal exhausted states [120]. Suppression of mTOR signaling in CAR-T cells not only increases CAR-T cell stemness but also increases CAR-T infiltration into bone marrow via CXCR4 expression and improves tumor cell elimination capacity of CAR-T cells [121,122]. Together, these studies paved a conceptual basis for reprogramming transcriptional networks by Rho GTPase signaling to alter CAR-T cell fate and function.

Concurrently, increasing attention has been directed toward the role of post-translational regulation in T cell anti-cancer activity, as evidenced by the generally weak correlation between RNA abundance and protein expression levels in exhausted cells [123]. Proteomic and genetic screens have identified protein turnover as critical regulators of T cell stemness and exhaustion [123–125]. In this context, AKT signaling, a downstream effector of Rho GTPases, induces proteotoxic stress responses in T cells to reinforce exhaustion [123] (Figure 3), raising the possibility that Rho GTPases may additionally regulate T cell fate through post-translational mechanisms.

Metabolic reprogramming is another central determinant of T cell exhaustion [126]. Specifically, glycolysis promotes CD8^+^ T cells toward terminal exhausted state [127]. Glycosphingolipids support CD8^+^ T cell anti-tumor activity [128,129], and cholesterol deficiency induces autophagy-mediated apoptosis and accelerates exhaustion [130]. In addition, loss of mitochondrial ‘fitness,’ including impaired oxidative phosphorylation, reduced fatty acid oxidation (FAO), and excessive mitochondrial stress, represents a hallmark of terminal T cell exhaustion [126,131]. These metabolic pathways have been shown to be regulated by Rho GTPases. As such, Rho GTPases are well studied to promote glycolysis [76], RAC1 enhances glycosphingolipid biosynthesis [12], RAC1 and RHOA regulate sterol regulatory element-binding proteins to control cholesterol biosynthesis, and CDC42 modulates cholesterol intracellular transportation and efflux [132–136]. For mitochondrial ‘fitness,’ ROCK inhibition increases expression of genes associated with FAO and mitochondrial energy production [137]. CDC42 also regulates mitochondrial fission and respiration [138], and RAC1 increases production of reactive oxygen species (ROS) through NADPH oxidase complexes and drives mitochondrial damage [139] (Figure 3). These studies provide hypothesis-generating evidence for Rho GTPase regulation of CAR-T exhaustion via metabolic reprogramming.

T cell aging may predispose T cells to exhaustion, whereas exhaustion can accelerate an aging-like program, collectively limiting CAR-T cell function [140]. The characteristics of T cell aging include a decrease in naïve T cells, an increase in memory, exhausted and senescent T cells, elevated inflammatory cytokines, impaired autophagy, mitochondrial dysfunction, increased ROS, and epigenetic alteration [141,142]. As discussed above, Rho GTPases participate in T cell fate changes, cytokine production and secretion, mitochondrial regulation, ROS production, and chromatin remodeling. Besides, CDC42 controls spatial organization of autophagy in hematopoietic stem cells (HSCs) and promotes autophagy in neurons [143,144]. Consistent with these roles, multiple studies have directly linked Rho GTPases to aging. Elevated CDC42 activity drives aging phenotypes in mice, and pharmacological inhibition of CDC42 with CASIN rejuvenates hair follicle stem cells and intestinal stem cells by restoring Wnt signaling, reverses skin aging through reduced RPL4 expression, and rejuvenates HSCs by modifying H4K16ac polarity [145–149]. Similarly, targeting RHOA nuclear mechanoactivity has been shown to rejuvenate aged HSCs via chromatin remodeling [150]. Collectively, these findings suggest that Rho GTPases play critical roles in cell aging and lend support for the hypothesis that selective targeting abnormal Rho GTPase signaling in aged T cells may represent an opportunity to rejuvenate T cells and enhance CAR-T cell efficiency.

The above insights provide mostly inferential data so far but help establish a compelling conceptual framework for future study of Rho GTPases in CAR-T therapy. Current evidence implicates Rho GTPase signaling may exert long-term effects on T cell exhaustion and function through coordinated effects on TCR signaling, transcriptional regulation, post-translational proteostasis, metabolic reprogramming, and cellular aging. The mechanistic work that directly defines the roles of individual Rho GTPases in regulating CAR-T cell stemness, exhaustion, persistence, and anti-tumor efficiency remains limited and needs future investigation.

### Potential role of Rho GTPases in NK cell therapy

CAR-NK cell therapy has emerged as a promising alternative to CAR-T cell therapy with advantages in safety and allogeneic applicability, but it has yet to be approved by the FDA and is limited by variable efficiency and durability [151]. Multiple studies have identified Rho GTPases as regulators of NK cell (nature killer cell) functions. RAC1 functions as a positive regulator of NK cell-mediated killing by promoting NK cell migration, stable conjugate formation with target cells, and polarization of lytic granules toward the immunological synapse [152,153]. RAC1 also interacts with STAT3 to enhance its binding to the promoter region of NKG2D in NK cells, thereby increasing NKG2D surface expression and strengthening NK cell-mediated cytotoxicity [154]. CDC42 signaling regulates actin organization and directional secretion at the NK immunological synapse with oscillatory activity, and excessive CDC42-driven actin remodeling can contribute to impaired NK cell cytotoxicity [155,156]. Inhibition of CDC42 rejuvenates aged human NK cells and enhances NK cell cytotoxicity [157]. Furthermore, RHOA signaling acts as a negative regulator in NK cells, as it inhibits actin reorganization to destabilize the immunological synapse and suppress NK cell cytotoxicity [158]. These mechanistic insights raise the possibility that selective enhancement of RAC1 activity or targeting CDC42 or RHOA during CAR-NK engineering may serve as a potential strategy to fine-tune cytoskeletal dynamics and improve CAR-NK efficiency.

## Summary

Classical Rho GTPases are multifaceted regulatory nodules connecting tumor cells, immune cells, and immunotherapeutic response. Given the recently realized druggability of classical Rho GTPases and associated signaling, continued dissection of these pathways will not only advance fundamental understanding of cancer immunology but also implicate new opportunities to improve the efficiency, durability, and scope of cancer immunotherapy.

In ICI therapy, there are some causal evidence showing that tumor cell intrinsic Rho GTPase signaling regulates immune evasion by modulating checkpoint protein expression, interferon response, immune cell infiltration, and metabolic competition within the TME. In parallel, Rho GTPases in immune cells exert cell type-specific effects that can either promote or restrain anti-tumor immunity. The duality highlights both the therapeutic promise and the complexity of targeting Rho GTPase pathways in immunotherapy. In CAR-T cell therapy, there is limited evidence directly associating Rho GTPase signaling with CAR-T outcomes. While various studies have implicated Rho GTPases in chronic TCR signaling, transcriptional remodeling, and metabolic regulation, their role in T cell exhaustion and aging remains underexplored. Given that exhaustion and aging represent a major barrier to durable CAR-T efficiency, this knowledge gap represents a critical area for future investigation. Future studies will be essential to define how specific Rho GTPase signaling nodules can be harnessed to improve persistence, functionality, and durability of CAR-based therapies.

A challenge is that Rho GTPase signaling exhibits dual and context-dependent functions, acting in either tumor-promoting or tumor-suppressive roles depending on cell type, mutation status, and microenvironmental context. This complexity extends to immunotherapy, where Rho GTPase signaling may simultaneously enhance tumor immune evasion while supporting immune cell effector function. Therefore, rather than a uniform targeting strategy, selective and cell type-specific modulation of Rho GTPase pathways may be required to achieve therapeutic benefit. Looking forward to several opportunities that lie ahead. First, most studies of Rho GTPases rely heavily on cellular overexpression or systematic genetic deletion in animal models, which often do not fully recapitulate the endogenous and heterozygous alterations observed in patients. Future genetically precise studies that more accurately reflect clinically relevant perturbations, combined with examinations of patient-derived tumor samples for functional validation beyond correlative analyses, will be important. Second, a deeper mechanistic understanding of how Rho GTPase signaling leads to exhaustion and aging of T/NK cells and myeloid cells, particularly in the engineered CAR cells, is needed for rational therapeutic intervention. Third, given tumor context-dependent and sometimes opposing roles of Rho GTPases, their targeting may require cell type-specific and temporally controlled modulation rather than systemic inhibition to maximize therapeutic benefit while minimizing adverse effects. For example, in CAR-T therapy, Rho signaling pathway targeting during *ex vivo* manipulation may enhance T cell fitness and persistence. Finally, a bottleneck for ICI and CAR-T therapies is to tackle the tumor-immune cell barriers that hinder effective immune attack of the tumor cells, especially in solid tumors. To this end, targeting Rho GTPase signaling that are crucial for maintaining the tumor-ECM and immune cell boundaries and for immune cell chemotaxis toward tumor cells may promote immune cell infiltration.

## Perspectives

- Highlight the importance of the field: Rho GTPase signaling network acts as a regulatory nodal at the crossroads of tumor cell and immune cell regulation crucial for immunotherapy
- Summary of the current thinking: Individual Rho GTPases in tumor cells and various immune cells modulate immunotherapy responses through actomyosin-mediated chemotaxis, cell junctions, cell polarity, and gene/epigenetic networks, among other pathways.
- Future directions: Further understanding Rho GTPase signaling in cancer immunomodulation will reveal new opportunities to improve the efficiency, durability, and scope of cancer immunotherapy.

## Open Access

Open access for this article is provided via our Subscribe to Open programme, made possible through the support of our subscribing institutions. Learn more here: https://portlandpress.com/pages/open_access_options_and_prices#conditional.

## Abbreviations

CAR: chimera-antigen receptor
CTLs: CD8^+^ effector T cells/cytotoxic T cell lymphocytes
DC: dendritic cell
FAO: fatty acid oxidation
HSCs: hematopoietic stem cells
ICI: immune checkpoint inhibitor
IFN-γ: interferon-γ
NET: neutrophil extracellular trap
NK: nature killer
PAKs: p21-activated kinases
PD-L1: programmed death-ligand 1
ROCK: Rho-associated coiled-coil-containing protein kinase
ROS: reactive oxygen species
TCR: T cell receptor
TME: tumor microenvironment
Treg: regulatory T cell
